# Stability Analysis of Impulsive Control Systems with Finite and Infinite Delays

**DOI:** 10.1155/2014/932395

**Published:** 2014-02-27

**Authors:** Xuling Wang, Xiaodi Li, Gani Tr. Stamov

**Affiliations:** ^1^School of Mathematical Sciences, Shandong Normal University, Ji'nan 250014, China; ^2^Department of Mathematics, Technical University of Sofia, 8800 Sliven, Bulgaria

## Abstract

This paper studies impulsive control systems with finite and infinite delays. Several stability criteria are established by employing the largest and smallest eigenvalue of matrix. Our sufficient conditions are less restrictive than the ones in the earlier literature. Moreover, it is shown that by using impulsive control, the delay systems can be stabilized even if it contains no stable matrix. Finally, some numerical examples are discussed to illustrate the theoretical results.

## 1. Introduction

As is known, a well-developed theory of impulsive control systems has come into existence (see [1–8] and the references therein). To our knowledge, such systems are now recognized as an excellent source of models to simulate processes and phenomena observed in control fields, such as orbital transfer of satellite [5, 7], dosage supply in pharmacokinetics [9], stabilization, and synchronization in chaotic secure communication systems and other chaos systems [10, 11]. Moreover, in some cases, continuous control is impossible and only impulsive control can be used. For example, a central bank cannot change its interest rate every day in order to regulate the money supply in a financial market.

On the other hand, time delay [12–20] occurs in many physical systems. So it is natural to study impulsive control systems with delays. Also, significant progress has been made in the theory of impulsive control systems with finite or infinite delays. Yang and Xu [21] have presented several interesting criteria on robust stability of uncertain impulsive control systems with time-varying delay by employing a formula for the variation of parameters and estimating the Cauchy matrix. Liu [22] has established several criteria on asymptotic stability of impulsive control systems with time delay by using the method of Lyapunov functions and Lyapunov functionals. By using the Razumikhin technique and Lyapunov functions, a new criterion on the uniform asymptotic stability and global stability of impulsive infinite delay differential systems has been obtained in [23]. However, to the best of our knowledge, there is no criterion on stability for impulsive control systems with delays by employing the largest and smallest eigenvalue of matrix. Hence, techniques and methods for impulsive control systems should be further developed and explored. The aim of this paper is to establish stability criteria for impulsive control systems with finite and infinite delays by employing the largest and smallest eigenvalue matrix.

Inspired by the idea in [22] dealing with impulsive control systems, some stability criteria are established. The rest of this paper is organized as follows. In Section 2, we introduce some notations and definitions. Then in Section 3, several stability criteria of impulsive control systems with finite and infinite delays by employing the largest and smallest eigenvalue of matrix are obtained. Finally, in Section 4, the advantages of the theoretical results are illustrated by two numerical examples.

## 2. Preliminaries

Let ℝ denote the set of real numbers, ℝ_+_ the set of nonnegative real numbers, ℝ^*n*^ the *n*-dimensional real space, and *N* = 1,2,…. The impulse times *t*_*k*_ satisfy 0 ≤ *t*_0_ < *t*_1_ < ⋯<*t*_*k*_ < ⋯, lim⁡_*k*→+*∞*_ *t*_*k*_ = +*∞*. For any *t* ≥ *t*_0_ ≥ 0 > *α* ≥ −*∞*, let ∫_0_^+*∞*^ *h*(*u*)*x*(*t* − *u*)*du*, where *s* ∈ [*t* + *α*, *t*] is a Volterra type functional. In this case, when *α* = −*∞*, the interval [*t* + *α*, *t*] is understood to be replaced by (−*∞*, *t*].

Consider the delay control system

(1) $$\begin{array}{c} \dot{x}\left(t\right)=Ax\left(t\right)+Bx\left(t-r\right)+C\int_{0}^{r}h\left(u\right)x\left(t-u\right)du,\;t\neq t_{k},\\ y\left(t\right)=Ex\left(t\right),\;t\geq 0, \end{array}$$

where *x* ∈ ℝ^*n*^ is the state variable, *y* ∈ ℝ^*m*^ is the output variable, *r* > 0 is a delay constant, *A*, *B*, *C* ∈ ℝ^*n*×*n*^ and *E* ∈ ℝ^*m*×*n*^ are known constant matrices, *h*(*u*) ∈ *C*(ℝ^+^, ℝ), and ∫_0_^*r*^|*h*(*u*)|*du* < *∞*.

An impulsive control law of (1) can be presented in the form of the following control sequence {*t*_*k*_, *U*(*k*, *x*(*t*_*k*_^−^))} (refer the reader to [22]):

(2) $$\begin{array}{c} \Delta x\left(t_{k}\right)=x\left(t_{k}^{+}\right)-x\left(t_{k}^{-}\right)=U\left(k,x\left(t_{k}^{-}\right)\right),\;k\in N. \end{array}$$

Let *U*(*k*, *x*) = *B*_*k*_ *y*, *B*_*k*_ ∈ ℝ^*n*×*m*^, and *C*_*k*_ = *B*_*k*_ *E*. Then we obtain the impulsive control system with finite delays as follows:

(3) $$\begin{array}{c} \dot{x}\left(t\right)=Ax\left(t\right)+Bx\left(t-r\right)+C\int_{0}^{r}h\left(u\right)x\left(t-u\right)du,\;t\neq t_{k},\\ \Delta x\left(t_{k}\right)=x\left(t_{k}^{+}\right)-x\left(t_{k}^{-}\right)=C_{k}x\left(t_{k}^{-}\right),\;k\in N,\\ x\left(t\right)=\phi \left(t\right),\;-r\leq t\leq 0, \end{array}$$

where *ϕ* : [−*r*, 0] → ℝ^*n*^ is continuous.

Furthermore, we can investigate the following impulsive control system with infinite delays:

(4) x˙(t)=Ax(t)+Bx(t−r)+C∫0+∞h(u)x(t−u)du,t≠tk,Δx(tk)=x(tk+)−x(tk−)=Ckx(tk−), k∈N,x(t)=ϕ(t), α≤t≤0,

where *ϕ* : [*α*, 0] → ℝ^*n*^ is continuous and ∫_0_^+*∞*^|*h*(*u*)|*du* < *∞*.

For any matrix *A* ∈ ℝ^*n*×*n*^, *λ*_max⁡_(*A*) denotes the largest eigenvalue of *A* and similarly *λ*_min⁡_(*A*) denotes the smallest eigenvalue of *A*. The norm of matrix is $||A||=\sqrt{\lambda_{\max}(A^{T}A)}$. For a positive definite (symmetric) matrix *A* ∈ ℝ^*n*×*n*^, $\sqrt{A}$ denotes the square root of *A*, which is defined to be the unique positive definite matrix satisfying $\sqrt{A}\cdot \sqrt{A}=A$ (see [24]).

Let us define the following class of functions for later use:

(5) K1={g∈C(ℝ+,ℝ+) ∣ g(0)=0,g(s)>0  for  s>0};K2={g∈C(ℝ+,ℝ+) ∣ g(0)=0,g(s)>0  for  s>0,  g  is  nondecreasing  in  s}.

In order to prove our main results, we need the following definitions and lemmas.

**Definition 1 (see [22])**

A matrix *A* ∈ ℝ^*n*×*n*^ is stable (or Hurwitz) if the eigenvalues of the matrix *A* all have negative real parts and there is a unique positive definite matrix *P* ∈ ℝ^*n*×*n*^ that solves the Lyapunov equation

(6) $$\begin{array}{c} A^{T}P+PA=-Q, \end{array}$$

where *Q* is any *n* × *n* positive definite matrix.

**Definition 2 (see [22])**

A matrix *A* ∈ ℝ^*n*×*n*^ is unstable if the eigenvalues of the matrix *A* all have positive real parts and there is a unique positive definite matrix *P* ∈ ℝ^*n*×*n*^ that solves the Lyapunov equation

(7) $$\begin{array}{c} A^{T}P+PA=Q, \end{array}$$

where *Q* is any *n* × *n* positive definite matrix.

**Definition 3 (see [23])**

Assume that *x*(*t*) = *x*(*t*, *t*_0_, *ϕ*) is the solution of (3) and (4) through (*t*_0_, *ϕ*). Then the trivial solution of (3) and (4) is said to be

(*H*_1_) stable, if for any *t*_0_ ≥ 0 and *ɛ* > 0 there exists some *δ* = *δ*(*ɛ*, *t*_0_) > 0 such that ||*ϕ*|| < *δ* implies ||*x*(*t*, *t*_0_, *ϕ*)|| < *ɛ*, *t* ≥ *t*_0_;
(*H*_2_) uniformly stable, if the *δ* in (*H*_1_) is independent of *t*_0_;
(*H*_3_) uniformly asymptotically stable, if (*H*_2_) holds and there exists some *η* > 0 such that for any *ɛ* > 0 there exists some *T* = *T*(*ɛ*, *η*) > 0 such that *t*_0_ ≥ 0 and ||*ϕ*|| < *η* together imply ||*x*(*t*, *t*_0_, *ϕ*)|| < *ɛ*, *t* ≥ *t*_0_ + *T*.

**Lemma 4 (see [25])**

Assume that there exist functions *a*, *b*, *c* ∈ *K*_1_, *p* ∈ *PC*(ℝ_+_, ℝ_+_), and $g,\bar{g}\in K_{2}$, where $s\leq \bar{g}(s)<g(s)$ for *s* > 0. Suppose further that *V* : [−*r*, *∞*) × *S*(*ρ*) → ℝ_+_ is continuous on [−*r*, *t*_0_) × *S*(*ρ*) and on [*t*_*k*−1_, *t*_*k*_) × *S*(*ρ*) for *k* = 1,2,…, lim⁡_(*t*,*y*)→(*t*_*k*_^−^,*x*)_ *V*(*t*, *y*) = *V*(*t*_*k*_^−^, *x*) exists. Moreover, *V*, restricted to ℝ_+_ × *S*(*ρ*), is locally Lipschitz in *x* and the following conditions are satisfied:

- *b*(||*x*||) ≤ *V*(*t*, *x*) ≤ *a*(||*x*||) for all (*t*, *x*)∈[−*r*, *∞*) × *S*(*ρ*);
- *D* ^+^ *V*(*t*, *ψ*(0))≤−*p*(*t*)*c*(*V*(*t*, *ψ*(0))) for all *t* ≠ *t*_*k*_ in ℝ_+_ and *ψ* ∈ *PC*([−*r*, 0], *S*(*ρ*)), whenever *g*(*V*(*t*, *ψ*(0))) ≥ *V*(*t* + *s*, *ψ*(*s*)) for *s* ∈ [−*r*, 0];
- $V(t_{k},\psi (0)+I(t_{k},\psi ))\leq \bar{g}(V(t_{k}^{-},\psi (0)))$ for all (*t*_*k*_, *ψ*) ∈ ℝ_+_ × *PC*([−*r*, 0], *S*(*ρ*_1_)) for which *ψ*(0^−^) = *ψ*(0);
- *τ* = inf⁡_*k*∈*N*_{*t*_*k*_ − *t*_*k*−1_} > 0,  *M*_2_ = sup⁡_*q*>0_∫_*q*_^*g*(*q*)^(*ds*/*c*(*s*)) < *∞*, and *M*_1_ = inf⁡_*t*≥0_∫_*t*_^*t*+*τ*^ *p*(*s*)*ds* > *M*_2_.

Then the trivial solution of (3) is uniformly asymptotically stable.

**Lemma 5 (see [25])**

Assume that there exist functions *a*, *b*, *c* ∈ *K*_1_,  *p* ∈ *PC*(ℝ_+_, ℝ_+_), and *g* ∈ *K*_2_ and *V* : [−*r*, *∞*) × *S*(*ρ*) → ℝ_+_ is continuous on [−*r*, *t*_0_) × *S*(*ρ*) and on [*t*_*k*−1_, *t*_*k*_) × *S*(*ρ*) for *k* = 1,2,…, lim⁡_(*t*,*y*)→(*t*_*k*_^−^,*x*)_ *V*(*t*, *y*) = *V*(*t*_*k*_^−^, *x*) exists. Moreover, *V*, restricted to ℝ_+_ × *S*(*ρ*), is locally Lipschitz in *x* and the following conditions are satisfied:

- *b*(||*x*||) ≤ *V*(*t*, *x*) ≤ *a*(||*x*||) for all (*t*, *x*)∈[−*r*, *∞*) × *S*(*ρ*);
- *D* ^+^ *V*(*t*, *ψ*(0)) ≤ *p*(*t*)*c*(*V*(*t*, *ψ*(0))) for all *t* ≠ *t*_*k*_ in ℝ_+_ and *ψ* ∈ *PC*([−*r*, 0], *S*(*ρ*)), whenever *V*(*t*, *ψ*(0)) ≥ *g*(*V*(*t* + *s*, *ψ*(*s*))) for *s* ∈ [−*r*, 0];
- *V*(*t*_*k*_, *ψ*(0) + *I*(*t*_*k*_, *ψ*)) ≤ *g*(*V*(*t*_*k*_^−^, *ψ*(0))) for all (*t*_*k*_, *ψ*) ∈ ℝ_+_ × *PC*([−*r*, 0], *S*(*ρ*_1_)) for which *ψ*(0^−^) = *ψ*(0);
- *τ* = sup⁡_*k*∈*N*_{*t*_*k*_ − *t*_*k*−1_} < *∞*, *M*_1_ = sup⁡_*t*≥0_∫_*t*_^*t*+*τ*^ *p*(*s*)*ds* < *∞*, and *M*_2_ = inf⁡_*q*>0_∫_*g*(*q*)_^*q*^(*ds*/*c*(*s*)) > *M*_1_.

Then the trivial solution of (3) is uniformly asymptotically stable.

**Lemma 6 (see [26])**

Assume that there exist functions *a*, *b*, *c* ∈ *K*_1_,  *p* ∈ *PC*(ℝ_+_, ℝ_+_), and *g* ∈ *K*_2_ and *V* : [*α*, *∞*) × *S*(*ρ*) → ℝ_+_ is continuous on [*α*, *t*_0_) × *S*(*ρ*) and on [*t*_*k*−1_, *t*_*k*_) × *S*(*ρ*) for *k* = 1,2,…, lim⁡_(*t*,*y*)→(*t*_*k*_^−^,*x*)_ *V*(*t*, *y*) = *V*(*t*_*k*_^−^, *x*) exists. Moreover, *V*, restricted to ℝ_+_ × *S*(*ρ*), is locally Lipschitz in *x* and the following conditions are satisfied:

- *b*(||*x*||) ≤ *V*(*t*, *x*) ≤ *a*(||*x*||) for all (*t*, *x*)∈[*α*, *∞*) × *S*(*ρ*);
- *D* ^+^ *V*(*t*, *x*) ≤ *p*(*t*)*c*(*V*(*t*, *x*)) for any solution *x*(*t*) = *x*(*t*, *t*_0_, *ϕ*) of (4), whenever *V*(*t*, *x*(*t*)) ≥ *g*(*V*(*s*, *x*(*s*))) for *s* ∈ [*α*, *t*];
- *V*(*t*_*k*_, *x* + *I*(*t*_*k*_, *x*)) ≤ *g*(*V*(*t*_*k*_^−^, *x*)) for all *k* ∈ *N* and all *x* ∈ *S*(*ρ*_1_);
- *M*_1_ = sup⁡_*k*∈*N*_∫_*t*_*k*__^*t*_*k*+1_^ *p*(*s*)*ds* < *∞* and *M*_2_ = inf⁡_*u*>0_∫_*g*(*u*)_^*u*^(*ds*/*c*(*s*)) > *M*_1_.

Then the trivial solution of (4) is uniformly stable.

## 3. Main Results

In this section, we will establish some stability criteria for system (3) and (4). Our first theorem provides conditions for uniform asymptotic stability of the trivial solution of (3), when matrix *A* is Hurwitz.

**Theorem 7**

Let *τ* = inf⁡_*k*∈*N*_{*t*_*k*_ − *t*_*k*−1_} > 0 and assume all the eigenvalues of *A* have negative real parts. Suppose that there exist a constant *γ* ≥ 1 and two symmetrical positive definite matrices *P*, *Q* ∈ ℝ^*n*×*n*^ such that

(8) $$\begin{array}{c} A^{T}P+PA=-Q,\\ \sqrt{\frac{\lambda_{\max}\left(P\right)}{\lambda_{\min}\left(P\right)}}||I+C_{k}||\leq \gamma ,\\ 2\gamma ||PB||+2\gamma 𝕄||PC||<\frac{\lambda_{\min}\left(Q\right)\lambda_{\min}\left(P\right)}{\lambda_{\max}\left(P\right)},\\ \begin{array}{ccc} \frac{\ln\gamma }{\tau }-\frac{\lambda_{\min}\left(Q\right)}{2\lambda_{\max}\left(P\right)}+\frac{\gamma ||PB||}{\lambda_{\min}\left(P\right)}+\frac{\gamma 𝕄||PC||}{\lambda_{\min}\left(P\right)}<0, & & \end{array} \end{array}$$

where *𝕄* = ∫_0_^*γ*^|*h*(*u*)|*du*. Then the trivial solution of (3) is uniformly asymptotically stable.

**Proof**

Define the Lyapunov function

(9) $$\begin{array}{c} V\left(x\right)=x^{T}Px. \end{array}$$

Then, *V* satisfies

(10) $$\begin{array}{c} \lambda_{\min}\left(P\right){||x||}^{2}\leq V\left(x\right)\leq \lambda_{\max}\left(P\right){||x||}^{2}, \end{array}$$

for all *x* ∈ ℝ^*n*^. Furthermore,

(11) V(ψ(0)+Ckψ(0)) =[(I+Ck)ψ(0)]TP[(I+Ck)ψ(0)] =ψ(0)T(I+Ck)TP(I+Ck)ψ(0) ≤λmax⁡(P)[(I+Ck)ψ(0)]T[(I+Ck)ψ(0)] ≤λmax⁡(P)||(I+Ck)ψ(0)||2 ≤λmax⁡(P)||(I+Ck)||2·||ψ(0)||2 ≤λmax⁡(P)λmin⁡(P)||I+Ck||2ψ(0)TPψ(0) ≤γ2V(ψ(0)).

Since *γ* satisfies the strict inequality (8), then both equalities are satisfied by any *β* chosen sufficiently close to *γ*. So let *β* > *γ* satisfy

(12) $$\begin{array}{c} 2\beta ||PB||+2\beta 𝕄||PC||<\frac{\lambda_{\min}\left(Q\right)\lambda_{\min}\left(P\right)}{\lambda_{\max}\left(P\right)}, \end{array}$$

(13) $$\begin{array}{c} \frac{\ln\beta }{\tau }-\frac{\lambda_{\min}\left(Q\right)}{2\lambda_{\max}\left(P\right)}+\frac{\beta ||PB||}{\lambda_{\min}\left(P\right)}+\frac{\beta 𝕄||PC||}{\lambda_{\min}\left(P\right)}<0. \end{array}$$

Define *g*(*s*) = *β* ^2^ *s* and $\bar{g}(s)=\gamma^{2}s$. Then, when *g*(*V*(*ψ*(0))) ≥ *V*(*ψ*(*s*)), *s* ∈ [−*r*, 0], so we have *β* ^2^ *V*(*ψ*(0)) ≥ *V*(*ψ*(−*r*)) and *β* ^2^ *V*(*ψ*(0)) ≥ *V*(*ψ*(−*u*)). Calculating the derivative of *V* along solutions of (3) gives us

(14) D+V(t,ψ) =ψ(0)TP[Aψ(0)+Bψ(−r)+ C∫0rh(u)ψ(−u)du]  +[Aψ(0)+Bψ(−r)+ C∫0rh(u)ψ(−u)du]TPψ(0) =ψ(0)T(ATP+PA)ψ(0)+2ψ(0)TPBψ(−r)  +2ψ(0)TPC∫0rh(u)ψ(−u)du ≤−λmin⁡(Q)||ψ(0)||2+2||PB||·||ψ(0)||·||ψ(−r)||  +2||PC||·||ψ(0)||∫0r|h(u)|·||ψ(−u)||du ≤−λmin⁡(Q)λmax⁡(P)ψ(0)TPψ(0)  +2||PB||λmin⁡(P)ψ(0)TPψ(0)·ψ(−r)TPψ(−r)  +2||PC||λmin⁡(P)ψ(0)TPψ(0)  ×∫0r|h(u)|ψ(−u)TPψ(−u) du ≤−λmin⁡(Q)λmax⁡(P)V(ψ(0))  +2||PB||λmin⁡(P)V(ψ(0))·V(ψ(−r))  +2||PC||λmin⁡(P)V(ψ(0))∫0r|h(u)|V(ψ(−u)) du ≤−λmin⁡(Q)λmax⁡(P)V(ψ(0))+2β||PB||λmin⁡(P)V(ψ(0))  +2β||PC||λmin⁡(P)∫0r|h(u)|duV(ψ(0)) ≤−(λmin⁡(Q)λmax⁡(P)−2β||PB||λmin⁡(P)−2β𝕄||PC||λmin⁡(P))V(ψ(0)).

Let *M*_1_ and *M*_2_ be given by

(15) $$\begin{array}{c} M_{1}=\frac{\tau \lambda_{\min}\left(Q\right)}{\lambda_{\max}\left(P\right)}-\frac{2\tau \beta ||PB||}{\lambda_{\min}\left(P\right)}-\frac{2\tau \beta 𝕄||PC||}{\lambda_{\min}\left(P\right)},\\ M_{2}=2\ln\beta . \end{array}$$

Then we have *M*_1_ > *M*_2_, by equality (13) on *β*. Thus, by Lemma 4, it follows that the trivial solution of (3) is uniformly asymptotically stable.

Our next theorem establishes conditions for uniform asymptotic stability of trivial solution of (3) when matrix *A* is unstable.

**Theorem 8**

Let *τ* = sup⁡_*k*∈*N*_{*t*_*k*_ − *t*_*k*−1_} < *∞* and assume all the eigenvalues of *A* have positive real parts. Suppose that there exist a constant 0 < *γ* < 1 and two symmetrical positive definite matrices *P*, *Q* ∈ ℝ^*n*×*n*^ such that

(16) $$\begin{array}{c} A^{T}P+PA=Q,\\ \sqrt{\frac{\lambda_{\max}\left(P\right)}{\lambda_{\min}\left(P\right)}}||I+C_{k}||\leq \gamma ,\\ \frac{\ln\gamma }{\tau }+\frac{\lambda_{\max}\left(Q\right)}{2\lambda_{\min}\left(P\right)}+\frac{||PB||}{\gamma \lambda_{\min}\left(P\right)}+\frac{𝕄||PC||}{\gamma \lambda_{\min}\left(P\right)}<0, \end{array}$$

where *𝕄* = ∫_0_^*γ*^|*h*(*u*)|*du*. Then the trivial solution of (3) is uniformly asymptotically stable.

**Proof**

Define the Lyapunov function

(17) $$\begin{array}{c} V\left(x\right)=x^{T}Px. \end{array}$$

Then, *V* satisfies

(18) $$\begin{array}{c} \lambda_{\min}\left(P\right){||x||}^{2}\leq V\left(x\right)\leq \lambda_{\max}\left(P\right){||x||}^{2}, \end{array}$$

for all *x* ∈ ℝ^*n*^. Furthermore,

(19) V(ψ(0)+Ckψ(0)) =[(I+Ck)ψ(0)]TP[(I+Ck)ψ(0)] =ψ(0)T(I+Ck)TP(I+Ck)ψ(0) ≤λmax⁡(P)[(I+Ck)ψ(0)]T[(I+Ck)ψ(0)] ≤λmax⁡(P)||(I+Ck)ψ(0)||2 ≤λmax⁡(P)||(I+Ck)||2·||ψ(0)||2 ≤λmax⁡(P)λmin⁡(P)||I+Ck||2ψ(0)TPψ(0)≤γ2V(ψ(0)).

Define *g*(*s*) = *γ* ^2^ *s*. Then if *V*(*ψ*(0)) ≥ *g*(*V*(*ψ*(*s*))), for *s* ∈ [−*r*, 0], we have *V*(*ψ*(−*r*)) ≤ *V*(*ψ*(0))/*γ* ^2^ and *V*(*ψ*(−*u*)) ≤ *V*(*ψ*(0))/*γ* ^2^. Calculating the derivative of *V* along solutions of (3) gives us

(20) D+V(t,ψ) =ψ(0)TP[Aψ(0)+Bψ(−r)+ C∫0rh(u)ψ(−u)du]  +[Aψ(0)+Bψ(−r)+C∫0rh(u)ψ(−u)du]TPψ(0)=ψ(0)T(ATP+PA)ψ(0)+2ψ(0)TPBψ(−r)+2ψ(0)TPC∫0rh(u)ψ(−u)du≤λmax⁡(Q)||ψ(0)||2+2||PB||·||ψ(0)||·||ψ(−r)||+2||PC||·||ψ(0)||∫0r|h(u)|·||ψ(−u)||du≤λmax⁡(Q)λmin⁡(P)ψ(0)TPψ(0)+2||PB||λmin⁡(P)ψ(0)TPψ(0)·ψ(−r)TPψ(−r)+2||PC||λmin⁡(P)ψ(0)TPψ(0)×∫0r|h(u)|ψ(−u)TPψ(−u) du≤λmax⁡(Q)λmin⁡(P)V(ψ(0))+2||PB||λmin⁡(P)V(ψ(0))·V(ψ(−r))+2||PC||λmin⁡(P)V(ψ(0))∫0r|h(u)|V(ψ(−u)) du≤λmax⁡(Q)λmin⁡(P)V(ψ(0))+2||PB||γλmin⁡(P)V(ψ(0))+2||PC||γλmin⁡(P)∫0r|h(u)|du V(ψ(0))≤(λmax⁡(Q)λmin⁡(P)+2||PB||γλmin⁡(P)+2𝕄||PC||γλmin⁡(P))V(ψ(0)).

Let *M*_1_ and *M*_2_ be given by

(21) $$\begin{array}{c} M_{1}=\frac{\tau \lambda_{\max}\left(Q\right)}{\lambda_{\min}\left(P\right)}+\frac{2\tau ||PB||}{\gamma \lambda_{\min}\left(P\right)}+\frac{2\tau 𝕄||PC||}{\gamma \lambda_{\min}\left(P\right)},\\ M_{2}=-2\ln\gamma . \end{array}$$

Then, we have *M*_2_ > *M*_1_, by inequality (16) on *γ*. Thus, by Lemma 5, it follows that the trivial solution of (3) is uniformly asymptotically stable.

**Remark 9**

Note that in Theorems 7 and 8, the criteria require the assumption that the time delay *γ* is finite, and so they may not be user-friendly in applications. Therefore, it is necessary to investigate the stability of impulsive control systems with infinite delays.

**Theorem 10**

Let *t*_*k*_ − *t*_*k*−1_ ≤ *μ* and assume that all the eigenvalues of *A* have positive real parts. Suppose that there exist a constant 0 < *γ* < 1 and two symmetrical positive definite matrices *P*, *Q* ∈ ℝ^*n*×*n*^ such that

(22) $$\begin{array}{c} A^{T}P+PA=Q,\\ \sqrt{\frac{\lambda_{\max}\left(P\right)}{\lambda_{\min}\left(P\right)}}||I+C_{k}||\leq \gamma ,\\ \frac{\ln\gamma }{\mu }+\frac{\lambda_{\max}\left(Q\right)}{2\lambda_{\min}\left(P\right)}+\frac{||PB||}{\gamma \lambda_{\min}\left(P\right)}+\frac{\mathbb{N}||PC||}{\gamma \lambda_{\min}\left(P\right)}<0, \end{array}$$

where *ℕ* = ∫_0_^+*∞*^|*h*(*u*)|*du*. Then the trivial solution of (4) is uniformly stable.

**Proof**

Define the Lyapunov function

(23) $$\begin{array}{c} V\left(x\right)=x^{T}Px. \end{array}$$

Then, *V* satisfies

(24) $$\begin{array}{c} \lambda_{\min}\left(P\right){||x||}^{2}\leq V\left(x\right)\leq \lambda_{\max}\left(P\right){||x||}^{2}, \end{array}$$

for all *x* ∈ ℝ^*n*^. Furthermore,

(25) V(ψ(0)+Ckψ(0)) =[(I+Ck)ψ(0)]TP[(I+Ck)ψ(0)] =ψ(0)T(I+Ck)TP(I+Ck)ψ(0) ≤λmax⁡(P)[(I+Ck)ψ(0)]T[(I+Ck)ψ(0)] =λmax⁡(P)||(I+Ck)ψ(0)||2 ≤λmax⁡(P)||(I+Ck)||2·||ψ(0)||2 ≤λmax⁡⁡(P)λmin⁡(P)||I+Ck||2ψ(0)TPψ(0)≤γ2V(ψ(0)).

Define *g*(*s*) = *γ* ^2^ *s*. Then if *V*(*ψ*(0)) ≥ *g*(*V*(*ψ*(*s*))), for *s* ∈ [−*∞*, 0], we have *V*(*ψ*(−*r*)) ≤ *V*(*ψ*(0))/*γ* ^2^ and *V*(*ψ*(−*u*)) ≤ *V*(*ψ*(0))/*γ* ^2^. Calculating the derivative of *V* along solutions of (3) gives us

(26) D+V(t,ψ) =ψ(0)TP[Aψ(0)+Bψ(−r)+ C∫0+∞h(u)ψ(−u)du]  +[Aψ(0)+Bψ(−r)+ C∫0+∞h(u)ψ(−u)du]TPψ(0) =ψ(0)T(ATP+PA)ψ(0)+2ψ(0)TPBψ(−r)  +2ψ(0)TPC∫0+∞h(u)ψ(−u)du ≤λmax⁡(Q)||ψ(0)||2+2||PB||·||ψ(0)||·||ψ(−r)||  +2||PC||·||ψ(0)||∫0+∞|h(u)|·||ψ(−u)||du ≤λmax⁡(Q)λmin⁡(P)ψ(0)TPψ(0)  +2||PB||λmin⁡(P)ψ(0)TPψ(0)·ψ(−r)TPψ(−r)  +2||PC||λmin⁡(P)ψ(0)TPψ(0)  ×∫0+∞|h(u)|ψ(−u)TPψ(−u) du ≤λmax⁡(Q)λmin⁡(P)V(ψ(0))+2||PB||λmin⁡(P)V(ψ(0))·V(ψ(−r))  +2||PC||λmin⁡(P)V(ψ(0))∫0+∞|h(u)|V(ψ(−u)) du ≤λmax⁡(Q)λmin⁡(P)V(ψ(0))+2||PB||γλmin⁡(P)V(ψ(0))  +2||PC||γλmin⁡(P)∫0+∞|h(u)|du V(ψ(0)) ≤(λmax⁡(Q)λmin⁡(P)+2||PB||γλmin⁡(P)+2ℕ||PC||γλmin⁡(P))V(ψ(0)).

Let *M*_1_ and *M*_2_ be given by

(27) $$\begin{array}{c} M_{1}=\frac{\mu \lambda_{\max}\left(Q\right)}{\lambda_{\min}\left(P\right)}+\frac{2\mu ||PB||}{\gamma \lambda_{\min}\left(P\right)}+\frac{2\mu \mathbb{N}||PC||}{\gamma \lambda_{\min}\left(P\right)},\\ M_{2}=-2\ln\gamma . \end{array}$$

Then, we have *M*_2_ > *M*_1_, by inequality (22) on *γ*. Thus, by Lemma 6, it follows that the trivial solution of (4) is uniformly stable.

**Remark 11**

In this paper, a new technique is offered to establish the stability criteria for finite and infinite delay impulsive control systems. In the above theories, rather than employing Razumikhin techniques and Lyapunov functions, we adopt the largest and smallest eigenvalue so that not only can they be easier constructed, but also the conditions ensuring the required stability are less restrictive. Furthermore, in Theorem 10, the obtained stability criterion is for impulsive control systems with both finite and infinite delays. Therefore, the result is rather general and has great power in applications.

## 4. Applications

**Example 1**

Consider the following impulsive control systems:

(28) x˙(t)=Ax(t)+Bx(t−r)+C∫0+∞h(u)x(t−u)du,t≠tk,Δx(tk)=x(tk+)−x(tk−)=Ckx(tk−), k∈N,

where *h*(*u*) = *e* ^−*u*^,  *u* > 0 and with the following parameter matrices:

(29) $$\begin{array}{c} A=\left[\begin{array}{cc} 1.2 & -1.1\\ 0.7 & 0.8 \end{array}\right],\;\;B=\left[\begin{array}{cc} -1.3 & 0.7\\ -0.9 & 0.5 \end{array}\right],\\ C=\left[\begin{array}{cc} -0.1 & -0.22\\ 0.43 & 0.65 \end{array}\right],\;\;C_{k}=\left[\begin{array}{cc} -0.8 & 0.2\\ -0.2 & -0.9 \end{array}\right]. \end{array}$$

**Property 1**

The trivial solution of the system (28) is uniformly stable if there exists constant *γ* ∈ (0.4922,1) such that

(30) $$\begin{array}{c} \frac{4.1189}{\gamma }+\frac{\ln\gamma }{\mu }<-2.8015. \end{array}$$

**Proof**

Obviously,

(31) $$\begin{array}{c} \mathbb{N}=\int_{0}^{+\infty }e^{-u}du=1. \end{array}$$

Choose a symmetric positive definite matrix *P* as follows:

(32) $$\begin{array}{c} P=\left[\begin{array}{cc} 0.16 & -0.08\\ -0.08 & 0.29 \end{array}\right]. \end{array}$$

We directly calculate the following parameters:

(33) $$\begin{array}{c} ||PB||=0.2371,\;\;||PC||=0.2650,\\ ||I+C_{k}||=0.300,\;\;\lambda_{\min}\left(P\right)=0.1219,\\ \lambda_{\max}\left(P\right)=0.3281,\;\;\sqrt{\frac{\lambda_{\max}\left(P\right)}{\lambda_{\min}\left(P\right)}}||I+C_{k}||=0.4922,\\ \lambda_{\max}\left(Q\right)=0.6830, \end{array}$$

where *Q* = *A* ^*T*^ *P* + *PA*. So by (30), it can be deduced that

(34) ln⁡γμ+λmax⁡(Q)2λmin⁡(P)+||PB||2γλmin⁡(P)+ℕ||PC||2γλmin⁡(P) =4.1189γ+ln⁡γμ+2.8015<0.

Suppose in addition that the impulsive times occur with a frequency of *μ* = 0.04. Also, a simple check shows that both of these conditions are satisfied by choosing *γ* = 0.5. Therefore, uniformly stable of the trivial solution of (28) can be obtained by Theorem 10.

**Example 2**

Consider the impulsive control systems (28) where *h*(*u*) = 2*e* ^−*u*^, *u* > 0 and with the following parameter matrices:

(35) $$\begin{array}{c} A=\left[\begin{array}{ccc} -2 & 1 & 1\\ 0 & 3 & -1\\ 1 & 2 & 4 \end{array}\right],\;\;\;B=\left[\begin{array}{ccc} 2 & 0 & 1\\ -1 & 1 & -2\\ -3 & 0 & 1 \end{array}\right],\\ C=\left[\begin{array}{ccc} 1 & -2 & 2\\ -2 & -1 & -1\\ 5 & -1 & 1 \end{array}\right],\;\;C_{k}=\left[\begin{array}{ccc} -1.1 & 0.1 & 0\\ 0 & -1.1 & 0.1\\ 0.2 & 0 & -1.2 \end{array}\right]. \end{array}$$

**Property 2**

The trivial solution of the system (28) is uniformly stable if there exists constant *γ* ∈ (0.5801,1) such that

(36) $$\begin{array}{c} \frac{26.6491}{\gamma }+\frac{\ln\gamma }{\mu }<-9.2250. \end{array}$$

**Proof**

Obviously,

(37) $$\begin{array}{c} \mathbb{N}=\int_{0}^{+\infty }2e^{-u}du=2. \end{array}$$

Choose a symmetric positive definite matrix *P* as follows:

(38) $$\begin{array}{c} P=\left[\begin{array}{ccc} 0.1929 & -0.0893 & -0.0786\\ -0.0893 & 0.2500 & 0.0714\\ -0.0786 & 0.0714 & 0.1643 \end{array}\right]. \end{array}$$

We directly calculate the following parameters:

(39) $$\begin{array}{c} {||PB||}_{2}=1.3240,\;\;{||PC||}_{2}=0.6518,\\ {||I+C_{k}||}_{2}=0.3000,\;\;\lambda_{\min}\left(P\right)=0.0986,\\ \lambda_{\max}\left(P\right)=0.3687,\;\;\sqrt{\frac{\lambda_{\max}\left(P\right)}{\lambda_{\min}\left(P\right)}}{||I+C_{k}||}_{2}=0.5801,\\ \lambda_{\max}\left(Q\right)=1.8251, \end{array}$$

where *Q* = *A* ^*T*^ *P* + *PA*. So by (36), it can be deduced that

(40) ln⁡γμ+λmax⁡(Q)2λmin⁡(P)+||PB||2γλmin⁡(P)+ℕ||PC||2γλmin⁡(P) =26.6491γ+ln⁡γμ+9.2550<0.

Suppose in addition that the impulsive times occur with a frequency of *ρ* = 0.009. Also, a simple check shows that both of these conditions are satisfied by choosing *γ* = 0.6. Therefore, robust exponential stability of the trivial solution of (28) can be obtained by Theorem 10.
